# Supplementary material for: Ventilator-Associated Pneumonia in COVID-19 Patients Admitted in Intensive Care Units: Relapse, Therapeutic Failure and Attributable Mortality—A Multicentric Observational Study from the OutcomeRea Network
Source: J Clin Med. 2023 Feb 6;12(4):1298. doi: 10.3390/jcm12041298 (PMC9961155; doi:10.3390/jcm12041298)
Supplement: Supplementary file 1 [file jcm-12-01298-s001.zip › jcm-2135147-supplementary.pdf]

Supplement Figure S1. Flow diagram

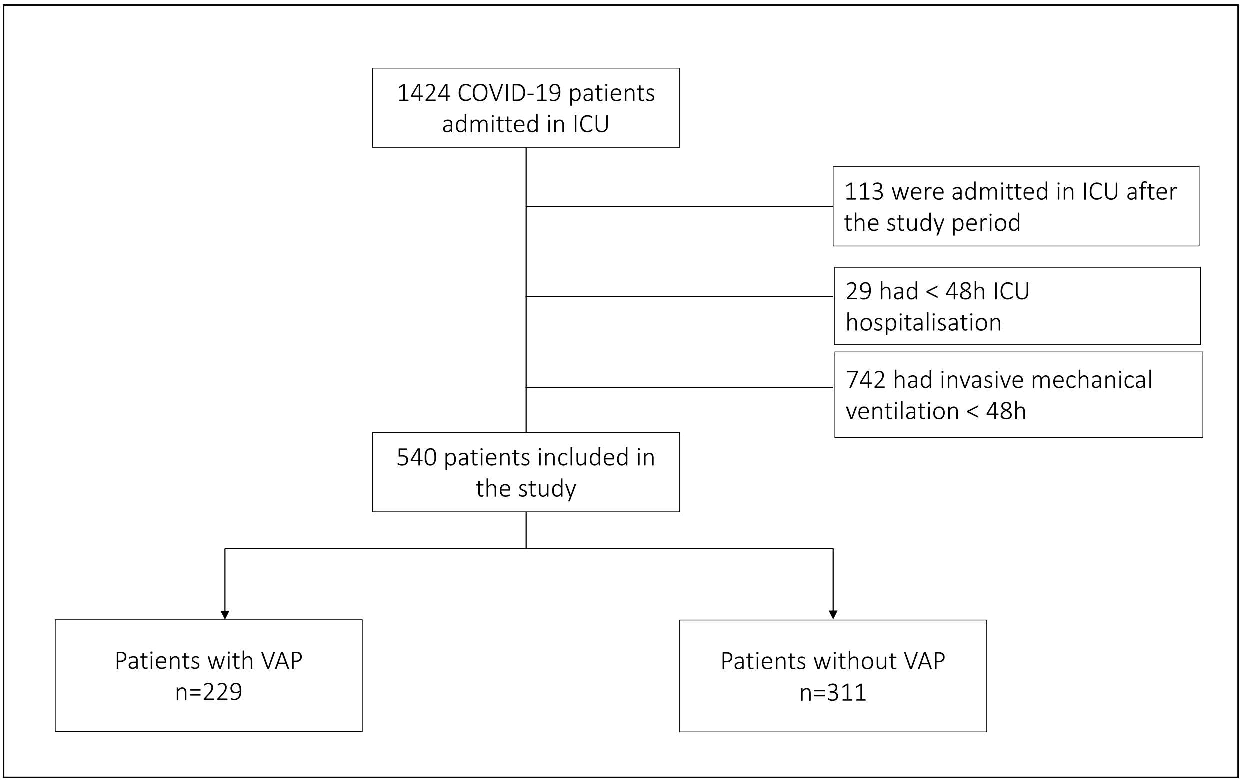

Supplement Table S1. Characteristics of pulmonary infections according to the timing and evolution under treatment (expressed by number of episodes of infections*:* 354 episodes of VAP occurred in 201 patients).

	VAP	Early VAP*	Late VAP*	*p*-value	1st VAP	Superinifection[†]	*p*-value	1st VAP	Relapse[‡]	*p*-value
Number of episodes	*n=383*	*n=95*	*n=288*		*n=236*	*n=73*		*n=230*	*n=105*	
Gram positive bacteria	124 (32.4)	37 (38.9)	87 (30.2)	0.11	81 (34.3)	24 (32.9)	0.82	78 (33.9)	31 (29.5)	0.43
Streptococcus pneumoniae	7 (1.8)	3 (3.2)	4 (1.4)	0.26	7 (3)	0 (0)	0.14	6 (2.6)	1 (1)	0.33
Other *Streptococci*	12 (3.2)	3 (3.2)	9 (3.1)	0.99	10 (4.2)	2 (2.7)	0.56	10 (4.3)	0 (0)	0.03
Staphylococcus aureus	84 (22)	25 (26.3)	59 (20.5)	0.23	50 (21.2)	20 (27.4)	0.27	49 (21.3)	24 (22.9)	0.75
Moraxella catarrhalis	1 (0.2)	0 (0)	1 (0.3)	0.57	1 (0.4)	0 (0)	0.58	1 (0.4)	0 (0)	0.50
Gram negative bacteria	316 (82.6)	72 (75.8)	244 (84.7)	0.05	185 (78.4)	60 (82.2)	0.48	180 (78.3)	92 (87.6)	0.04
Haemophilus influenza	16 (4.2)	10 (10.5)	6 (2.1)	< 0.01	16 (6.8)	0 (0)	0.02	16 (7)	0 (0)	< 0.01
Enterobacterales	191 (49.8)	37 (38.9)	154 (53.5)	0.01	121 (51.3)	33 (45.2)	0.37	118 (51.3)	50 (47.6)	0.53
Group 1 or 2 *enterobacterales*	103 (26.8)	21 (22.1)	82 (28.5)	0.22	68 (28.8)	21 (28.8)	0.99	67 (29.1)	21 (20)	0.08
Proteus sp	15 (4)	0 (0)	15 (5.2)	0.02	8 (3.4)	6 (8.2)	0.08	8 (3.5)	2 (1.9)	0.43
Escherichia coli	49 (12.8)	8 (8.4)	41 (14.2)	0.14	30 (12.7)	12 (16.4)	0.42	29 (12.6)	11 (10.5)	0.58
Klebsiella pneumoniae	42 (11)	12 (12.6)	30 (10.4)	0.55	30 (12.7)	6 (8.2)	0.30	30 (13)	9 (8.6)	0.24
Citrobacter koserii	6 (1.6)	2 (2.1)	4 (1.4)	0.63	5 (2.1)	1 (1.4)	0.69	5 (2.2)	1 (1)	0.43
Group 3 *enterobacterales*	88 (23)	11 (11.6)	55 (19.1)	0.09	38 (16.1)	10 (13.7)	0.62	36 (15.7)	25 (23.8)	0.07
Enterobacter sp	66 (17.2)	18 (18.9)	70 (24.3)	0.28	52 (22)	15 (20.5)	0.79	50 (21.7)	32 (30.5)	0.08
Serratia marcescens	16 (4.2)	5 (5.3)	11 (3.8)	0.54	11 (4.7)	4 (5.5)	0.78	11 (4.8)	4 (3.8)	0.69
Citrobacter freundii	3 (0.8)	1 (1.1)	2 (0.7)	0.73	1 (0.4)	0 (0)	0.58	1 (0.4)	2 (1.9)	0.19
Morganella morganii	5 (1.4)	1 (1.1)	4 (1.4)	0.80	4 (1.7)	1 (1.4)	0.85	4 (1.7)	1 (1)	0.58
Non-fermenting GNB	133 (34.8)	28 (29.5)	105 (36.5)	0.21	57 (24.2)	33 (45.2)	< 0.01	55 (23.9)	54 (51.4)	< 0.01
Pseudomonas aeruginosa	95 (24.8)	18 (18.9)	77 (26.7)	0.13	42 (17.8)	25 (34.2)	< 0.01	41 (17.8)	37 (35.2)	< 0.01
Stenotrophomonas maltophilia	17 (4.4)	5 (5.3)	12 (4.2)	0.65	9 (3.8)	1 (1.4)	0.30	9 (3.9)	7 (6.7)	0.27

Acinetobacter baumannii	24 (6.2)	5 (5.3)	19 (6.6)	0.64	6 (2.5)	11 (15.1)	< 0.01	5 (2.2)	12 (11.4)	< 0.01
MDR or XDR	114 (29.8)	18 (18.9)	96 (33.3)	< 0.01	64 (27.1)	23 (31.5)	0.47	62 (27)	38 (36.2)	0.09
ESBL-producing bacteria	52 (13.6)	12 (12.6)	40 (13.9)	0.76	33 (14)	9 (12.3)	0.72	32 (13.9)	14 (13.3)	0.89
Carbapenemase-producing bacteria	7 (1.8)	0 (0)	7 (2.4)	0.13	5 (2.1)	0 (0)	0.21	5 (2.2)	2 (1.9)	0.87
AmpC-hyperproduction	7 (1.8)	3 (3.2)	4 (1.4)	0.26	7 (3)	0 (0)	0.14	7 (3)	0 (0)	0.07
Resistant *Pseudomonas aeruginosa*§	36 (9.4)	4 (4.2)	32 (11.1)	0.05	12 (5.1)	8 (11)	0.07	12 (5.2)	21 (20)	< 0.01
Carbapenem Resistant *Acinetobacter baumannii*	11 (2.8)	2 (2.1)	9 (3.1)	0.61	2 (0.8)	7 (9.6)	< 0.01	2 (0.9)	4 (3.8)	0.06
MRSA	13 (3.4)	0 (0)	13 (4.5)	0.04	5 (2.1)	7 (9.6)	< 0.01	5 (2.2)	7 (6.7)	0.04
Susceptible species (*Streptooccus sp, Moraxella, Haemophilus*)	34 (8.8)	14 (14.7)	20 (6.9)	0.02	32 (13.6)	2 (2.7)	< 0.01	31 (13.5)	1 (1)	< 0.01
More than one strain	95 (24.8)	17 (17.9)	78 (27.1)	0.07	45 (19.1)	31 (42.5)	< 0.01	43 (18.7)	35 (33.3)	< 0.01
Time to adequate Antimicrobial therapy	1 [1 - 2]	1 [1 - 2]	1 [1 - 3]	0.70	1 [1 - 2]	2 [1 - 3]	0.27	1 [1 - 2]	1 [-3 - 2]	0.04
Adequacy	164 (42.8)	44 (46.3)	120 (41.7)	.	105 (44.7)	34 (48.6)	.	104 (45.4)	37 (35.9)	.
Bi antimicrobial therapy	159 (41.6)	44 (46.3)	115 (39.9)	0.27	92 (39)	30 (41.1)	0.75	88 (38.3)	49 (46.7)	0.15
Aminoside	72 (18.8)	18 (18.9)	54 (18.8)	0.97	46 (19.5)	17 (23.3)	0.48	45 (19.6)	14 (13.3)	0.16
Fluoroquinolone	53 (13.8)	14 (14.7)	39 (13.5)	0.77	25 (10.6)	8 (11)	0.93	24 (10.4)	24 (22.9)	< 0.01

*Early-onset VAP was defined as VAP occurring within the first 7 days after invasive ventilation, and late-onset VAP as VAP occurring on or after the 7th day after invasive ventilation.
§ Resistance to ticarcillin, ceftazidime or carbapenem
† different pathogen than previously isolated
‡ same pathogens as previously isolated
VAP: Ventilator-Associated Pneumonia; GNB: Gram Negative Bacteria; MDR: Multi-drug resistant; XDR: Extended-drug resistant; ESBL: Extended-spectrum ß-lactamase; MRSA: Methicillin-resistant *Staphylococcus aureus*.

Supplement Table S2. Risks factors associated with day-60 mortality (univariate analysis).

	Alive n=287	Dead n=253	*p*-value	Hazard Ratio	HR 95% CI	*p*-value
Period (before May 2020)	165 (57.5)	119 (47)	0.02	0.89	[0.67 - 1.18]	0.41
Age						
< 50	58 (20.2)	16 (6.3)	-	-	-	-
50 - 60	64 (22.3)	109 (43.1)	-	3.86	[2.26 - 6.61]	< 0.01
60 - 70	77 (26.8)	85 (33.6)	-	2.98	[1.74 - 5.12]	< 0.01
>70	88 (30.7)	43 (17)	< 0.01	1.48	[0.83 - 2.66]	0.18
Male gender	211 (73.5)	190 (75.1)	0.68	0.98	[0.73 - 1.32]	0.91
Body-mass index ≥ 30 kg/m^2	120 (41.8)	103 (40.7)	0.80	0.94	[0.72 - 1.21]	0.62
Comorbidities	165 (57.5)	192 (75.9)	< 0.01	1.70	[1.26 - 2.29]	< 0.01
Immunosuppression	16 (5.6)	42 (16.6)	< 0.01	1.61	[1.13 - 2.28]	0.01
ICU admission characteristics						
Time between symptoms and ICU admission > 10 days	110 (38.3)	74 (29.2)	0.03	0.74	[0.56 - 0.98]	0.04
Time between ICU admission and intubation > 5 days	16 (5.6)	51 (20.2)	< 0.01	2.08	[1.47 - 2.96]	< 0.01
MDR colonization	13 (4.5)	19 (7.5)	0.14	1.15	[0.7 - 1.9]	0.58
Corticosteroid	126 (43.9)	151 (59.7)	< 0.01	1.42	[1.08 - 1.87]	0.01
Immunomodulator	41 (14.3)	43 (17)	0.39	1.04	[0.73 - 1.47]	0.84
Antibiotic therapy	216 (75.3)	171 (67.6)	0.05	0.70	[0.53 - 0.92]	0.01
Broad spectrum antibiotics	26 (9.1)	44 (17.4)	< 0.01	1.44	[1.03 - 2.01]	0.03
Carbapenems	9 (3.1)	7 (2.8)	0.80	0.92	[0.38 - 2.25]	0.86
During the first 2 days of intubation						
PEEP > 12 cmH_2O	77 (26.8)	89 (35.2)	0.04	1.17	[0.89 - 1.53]	0.25
Prone position	98 (34.1)	77 (30.4)	0.36	0.90	[0.67 - 1.2]	0.48
Neuromuscular blockade	227 (79.1)	209 (82.6)	0.30	1.09	[0.77 - 1.55]	0.61
ECMO	21 (7.3)	26 (10.3)	0.22	0.98	[0.64 - 1.51]	0.94
Renal replacement therapy	16 (5.6)	40 (15.8)	< 0.01	1.60	[1.11 - 2.32]	0.01
Vasopressor	161 (56.1)	145 (57.3)	0.78	1.08	[0.78 - 1.5]	0.64
Enteral nutrition	187 (65.2)	145 (57.3)	0.06	0.65	[0.48 - 0.88]	0.01
Parenteral nutrition	36 (12.5)	70 (27.7)	< 0.01	1.88	[1.33 - 2.65]	< 0.01
Lymphocytes/neutrophils ratio>0.1	0.1 [0.1 - 0.1]	0.1 [0 - 0.1]	< 0.01	0.57	[0.41 - 0.79]	<.01
Before period at risk of VAP	`					
Antibiotic therapy	254 (88.5)	216 (85.4)	0.28	0.76	[0.53 - 1.09]	0.14
Broad spectrum antibiotic therapy	87 (30.3)	87 (34.4)	0.31	1.02	[0.78 - 1.34]	0.86
Carbapenems	29 (10.1)	19 (7.5)	0.29	0.63	[0.38 - 1.05]	0.08

Type of VAP						
VAP	120 (41.8)	109 (43.1)	0.77	1.25	[0.94 - 1.65]	0.12
Early VAP	45 (15.7)	48 (19)	0.31	1.03	[0.74 - 1.43]	0.88
Late VAP	99 (34.5)	80 (31.6)	0.48	1.36	[0.99 - 1.85]	0.06
Superinfection†	36 (12.5)	22 (8.7)	0.15	1.24	[0.73 - 2.1]	0.42
Relapse‡	38 (13.2)	24 (9.5)	0.17	1.20	[0.74 - 1.94]	0.45

† different pathogen than previously isolated
‡ same pathogens as previously isolated
ICU: Intensive care unit; MDR: Multi-drug Resistant; PEEP: Positive-End-Expiratory pressure; ECMO: Extracorporeal Membrane Oxygenation; VAP: Ventilator-Associated Pneumonia; ATB: Antibiotic

Supplement Table S3. Risk factors associated with superinfection (univariate analysis).

	No Superinfection n=172	Superinfection n=57	Hazard Ratio	HR95% IC	*p*-value
Period (before May 2020)	104 (60.5)	18 (31.6)	0.41	[0.23 - 0.72]	<0.01
Age > 70	55 (32)	13 (22.8)	0.71	[0.36 - 1.38]	0.31
Male gender	135 (78.5)	43 (75.4)	0.86	[0.48 - 1.53]	0.61
Body-mass index ≥ 30 kg/m²	72 (41.9)	34 (59.6)	1.69	[1 - 2.86]	0.05
Immunosuppression	10 (5.8)	4 (7)	1.09	[0.4 - 2.95]	0.87
Time between ICU admission and intubation > 5 days	15 (8.7)	8 (14)	1.14	[0.5 - 2.62]	0.76
Characteristics before the day of VAP					
Tocilizumab	8 (4.7)	4 (7)	1.72	[0.61 - 4.82]	0.30
Carbapenems	13 (7.6)	5 (8.8)	1.16	[0.45 - 2.99]	0.76
MDR colonization	18 (10.5)	7 (12.3)	1.19	[0.53 - 2.67]	0.67
Steroids	99 (57.6)	32 (56.1)	1.00	[0.6 - 1.68]	0.99
ECMO	20 (11.6)	17 (29.8)	2.30	[1.23 - 4.32]	0.01
PEEP > 12 cmH_2O	43 (25)	18 (31.6)	1.25	[0.7 - 2.25]	0.45
Prone position	50 (29.1)	17 (29.8)	1.00	[0.56 - 1.79]	1.00
Renal replacement therapy	38 (22.1)	11 (19.3)	0.80	[0.4 - 1.62]	0.54
Vasopressor	68 (39.5)	25 (43.9)	1.64	[0.86 - 3.15]	0.14
Enteral nutrition	133 (77.3)	45 (78.9)	0.92	[0.44 - 1.92]	0.83
Parenteral nutrition	53 (30.8)	18 (31.6)	1.39	[0.74 - 2.61]	0.31
Proto pump inhibitor	127 (73.8)	41 (71.9)	0.91	[0.49 - 1.71]	0.78
Lymphocytes/neutrophils ratio > 1	124 (72.1)	44 (77.2)	1.10	[0.57 - 2.11]	0.78
Highly susceptible germs	20 (11.6)	12 (21.1)	2.15	[1.18 - 3.93]	0.01
Staphylococcus aureus species	31 (18)	18 (31.6)	1.59	[0.91 - 2.78]	0.10
Group 1-2 *enterobacterales*	53 (30.8)	11 (19.3)	0.56	[0.28 - 1.12]	0.10
Group 3 *enterobacterales*	42 (24.4)	10 (17.5)	0.79	[0.39 - 1.58]	0.50
Non fermentant species	48 (27.9)	7 (12.3)	0.39	[0.18 - 0.84]	0.02
MDR or XDR	54 (31.4)	7 (12.3)	0.36	[0.16 - 0.8]	0.01
Bi antimicrobial therapy	73 (42.4)	15 (26.3)	0.51	[0.28 - 0.92]	0.03
Broad spectrum antimicrobial therapy	110 (64)	28 (49.1)	0.59	[0.35 - 1]	0.05
Adequation	81 (47.1)	29 (50.9)	1.21	[0.72 - 2.04]	0.47

ICU: Intensive care unit; VAP: Ventilator-Associated Pneumonia; ECMO: Extracorporeal Membrane Oxygenation; PEEP: Positive-End-Expiratory pressure; Non-fermenting species: *Pseudomonas aeruginosa*, *Acinetobacter baumannii*, *Stenotrophomonas maltophilia*; MDR: Multi-drug Resistant; XDR: Extended-drug resistant.

Supplement Table S4. Risk factors associated with relapse (univariate analysis).

	No Relapse n=168	Relapse n=61	Hazard Ratio	HR 95% IC	*p*-value
Period (before May 2020)	98 (58.3)	24 (39.3)	0.61	[0.36 - 1.03]	0.07
Age >70	46 (27.4)	16 (26.2)	0.70	[0.31 - 1.57]	0.39
Male gender	126 (75)	52 (85.2)	1.87	[0.94 - 3.75]	0.08
Body-mass index ≥ 30 kg/m²	77 (45.8)	29 (47.5)	1.00	[0.6 - 1.64]	0.99
Immunosuppression	12 (7.1)	2 (3.3)	0.39	[0.11 - 1.35]	0.14
Time between ICU admission and intubation > 5 days	15 (8.9)	8 (13.1)	1.13	[0.52 - 2.46]	0.75
Characteristics before the day of VAP					
Tocilizumab	9 (5.4)	3 (4.9)	1.27	[0.37 - 4.3]	0.70
Carbapenems	10 (6)	8 (13.1)	1.77	[0.85 - 3.68]	0.12
MDR colonization	16 (9.5)	9 (14.8)	1.27	[0.63 - 2.56]	0.50
Steroids	94 (56)	37 (60.7)	1.19	[0.72 - 1.99]	0.49
ECMO	19 (11.3)	18 (29.5)	1.69	[0.98 - 2.9]	0.06
PEEP > 12 cmH_2O	44 (26.2)	17 (27.9)	0.99	[0.54 - 1.83]	0.99
Prone position	45 (26.8)	22 (36.1)	1.27	[0.73 - 2.21]	0.39
Renal replacement therapy	38 (22.6)	11 (18)	0.65	[0.34 - 1.26]	0.20
Vasopressor	66 (39.3)	27 (44.3)	2.00	[1.14 - 3.51]	0.02
Enteral nutrition	125 (74.4)	53 (86.9)	1.15	[0.48 - 2.76]	0.76
Parenteral nutrition	58 (34.5)	13 (21.3)	1.24	[0.58 - 2.66]	0.58
Proto pump inhibitor	121 (72)	47 (77)	1.21	[0.64 - 2.28]	0.56
Lymphocytes/neutrophils ratio > 1	122 (72.6)	46 (75.4)	1.27	[0.71 - 2.27]	0.42
Highly susceptible germs	25 (14.9)	0 (0)			
Staphylococcus aureus species	36 (21.4)	8 (13.1)	0.57	[0.28 - 1.16]	0.12
Group 1-2 *enterobacteriacae*	45 (26.8)	16 (26.2)	1.02	[0.57 - 1.84]	0.94
Group 3 *enterobacteriacae*	37 (22)	18 (29.5)	1.47	[0.84 - 2.56]	0.17
Non fermentant species	38 (22.6)	23 (37.7)	1.54	[0.94 - 2.53]	0.08
MDR or XDR	45 (26.8)	16 (26.2)	1.06	[0.59 - 1.92]	0.83
Bi antimicrobial therapy	64 (38.1)	24 (39.3)	1.04	[0.62 - 1.74]	0.88
Broad spectrum antimicrobial therapy	97 (57.7)	41 (67.2)	1.48	[0.87 - 2.54]	0.15
Adequation	80 (47.6)	30 (49.2)	1.13	[0.69 - 1.86]	0.62

ICU: Intensive care unit; VAP: Ventilator-Associated Pneumonia; ECMO: Extracorporeal Membrane Oxygenation; PEEP: Positive-End-Expiratory pressure; Non-fermenting species: *Pseudomonas aeruginosa*, *Acinetobacter baumannii*, *Stenotrophomonas maltophilia*; MDR: Multi-drug Resistant; XDR: Extended-drug resistant.

Supplement Table S5. Risk factors associated with treatment failure (univariate analysis).

	No Failure n=72	Failure n=157	Hazard Ratio	HR 95% IC	*p*-value
Period of admission (before May 2020)	47 (65.3)	75 (47.8)	0.97	[0.69 - 1.36]	0.85
Age > 70 y	13 (18.1)	55 (35)	1.90	[1.33 - 2.72]	<0.01
Male gender	55 (76.4)	123 (78.3)	0.90	[0.6 - 1.33]	0.58
Body-mass index ≥ 30 kg/m²	31 (43.1)	75 (47.8)	1.10	[0.8 - 1.51]	0.55
Immunosuppression	3 (4.2)	11 (7)	0.77	[0.41 - 1.44]	0.42
Time between ICU admission and intubation > 5 days	1 (1.4)	22 (14)	1.30	[0.8 - 2.09]	0.28
Characteristics before the day of VAP					
Tocilizumab	4 (5.6)	8 (5.1)	1.30	[0.62 - 2.73]	0.48
Carbapenems	7 (9.7)	11 (7)	0.63	[0.34 - 1.18]	0.15
MDR colonization	8 (11.1)	17 (10.8)	1.09	[0.66 - 1.83]	0.73
Steroids	38 (52.8)	93 (59.2)	1.46	[1.05 - 2.03]	0.02
ECMO	2 (2.8)	35 (22.3)	0.99	[0.66 - 1.5]	0.98
PEEP > 12 cmH_2O	9 (12.5)	52 (33.1)	1.36	[0.94 - 1.96]	0.11
Prone position	16 (22.2)	51 (32.5)	1.22	[0.86 - 1.74]	0.26
Renal replacement therapy	6 (8.3)	43 (27.4)	1.42	[0.99 - 2.03]	0.06
Vasopressor	21 (29.2)	72 (45.9)	1.24	[0.85 - 1.82]	0.27
Enteral nutrition	60 (83.3)	118 (75.2)	0.82	[0.52 - 1.3]	0.40
Parenteral nutrition	19 (26.4)	52 (33.1)	1.53	[0.98 - 2.39]	0.06
Proto pump inhibitor	51 (70.8)	117 (74.5)	1.27	[0.85 - 1.88]	0.24
Lymphocytes/neutrophils ratio > 1	51 (70.8)	117 (74.5)	0.93	[0.63 - 1.37]	0.70
Highly susceptible germs	10 (13.9)	22 (14)	1.07	[0.68 - 1.7]	0.77
Staphylococcus aureus species	16 (22.2)	33 (21)	0.98	[0.66 - 1.46]	0.93
Group 1 or 2 *enterobacteriacae*	24 (33.3)	40 (25.5)	0.99	[0.68 - 1.45]	0.95
Group 3 *enterobacteriacae*	21 (29.2)	31 (19.7)	0.85	[0.57 - 1.27]	0.44
Non fermentant species	17 (23.6)	38 (24.2)	1.21	[0.83 - 1.77]	0.32
MDR or XDR	19 (26.4)	42 (26.8)	1.08	[0.75 - 1.56]	0.67
Bi antimicrobial therapy	23 (31.9)	65 (41.4)	1.07	[0.78 - 1.49]	0.67
Broad spectrum antimicrobial therapy	50 (69.4)	88 (56.1)	0.77	[0.55 - 1.08]	0.13
Adequation	32 (44.4)	78 (49.7)	1.09	[0.79 - 1.51]	0.60

ICU: Intensive care unit; VAP: Ventilator-Associated Pneumonia; ECMO: Extracorporeal Membrane Oxygenation; PEEP: Positive-End-Expiratory pressure; Non-fermenting species: *Pseudomonas aeruginosa, Acinetobacter baumannii, Stenotrophomonas maltophilia*; MDR: Multi-drug Resistant; XDR: Extended-drug resistant.

Supplement Table S6. Main recent studies describing VAP in COVID-19 patients.

Study	Sample size	VAP incidence †	VAP complications	Mortality‡
Rouzé et al	568	36 / NA	23% MDR	29.2%
Garcia-Vidal et al	144	25 / NA	-	9.8%
Pickens et al	179	44.4 / 45.2	-	19%
Gragueb-Chatti et al	151	60 / 26	37% recurrence,<br>68% with same pathogen	17% d-28,<br>26% d-60
Luyt et al	50	86 / NA	66% ≥ 1 recurrence,<br>38% polymicrobial	34%
Maes et al	81	48 / 28	-	38%
Blonz et al	188	48.9 / 33.7	20% multiple VAP,<br>3.6% empyema<br>1.4% abscesses	30.4% (VAP),<br>29% (overall)
Razazi et al	90	64 / NA	25% recurrence (23% MDR)	40%
Llitjos et al	176	52 / NA	21% recurrence	31%
Moretti et al	39	54 / NA	-	44%
Rouyer et al	79	53 / NA	28% recurrences,<br>17% clinical success (d-7)	52%
Giacobbe et al	586	29 / NA	-	46%
Contou et al	73	64 / NA	Among 73 death in ICU<br>23% recurrence (21% MDR)	48%
Grasselli et al	774	50 / 11.7	-	26%
D’Humières et al	77	84.4 / NA	57.4% failure at (d-7)	51% (overall)
Beaucoté et al	161	73 / NA	14% abscess, polymicrobial	56%, (65% if<br>lung abscess)

NA: not available; ECMO: Extra-Corporeal Membrane Oxygenation; MDR: Multi-drug Resistant.

† VAP crude incidence (%) and incidence density (expressed per 1000 mechanical ventilation days), whenever available.

‡ Crude ICU-mortality (%), d-28 or d-60 or attributable mortality whenever available.
